# Impact of Child Respiratory Syncytial Virus-Confirmed Infections on Caregivers in Japan: A Web-Based Survey

**DOI:** 10.3390/jcm13185355

**Published:** 2024-09-10

**Authors:** Mamoru Tanaka, Ichiro Morioka, Yu Funakoshi, Linghua Xu, Yasuhiro Kobayashi, Akari Muramatu, Shuhei Ito

**Affiliations:** 1 Department of Obstetrics and Gynecology, Keio University School of Medicine, Tokyo 160-8582, Japan; 2 Department of Pediatrics and Child Health, Nihon University School of Medicine, Tokyo 173-8610, Japan; 3 Pfizer Japan Inc., Tokyo 151-8589, Japan

**Keywords:** respiratory syncytial virus, parental burden, questionnaire survey, psychological impact, social impact, economic impact

## Abstract

**Background/Objectives:** Despite being the leading cause of acute lower respiratory tract infections in infants, the impact of respiratory syncytial virus (RSV) on the caregivers of infected children remains largely unexplored. This study is the first in Japan to examine the psychological, social, and economic burdens on caregivers of infants infected with RSV. **Methods:** An online questionnaire survey was used to understand the circumstances surrounding RSV infection and the psychological, social, and economic burdens on caregivers. Equal numbers of infants aged either <6 or ≥6 months were enrolled. **Results:** A total of 606 caregivers were included in the final analysis. Notably, 36.1% of the infants were hospitalized. Most caregivers (91.4%) felt anxious about their infants’ RSV infection, and more than half (55.8%) answered that their anxiety interfered with their daily lives. Caregivers whose daily routines were disrupted due to concerns about RSV infection were more likely to hospitalize infants, particularly for extended stays. Infection significantly affected family dynamics, hindering normal daily activities and escalating stress, which in turn led to conflicts and arguments among family members (30.4%). Regarding the financial burden, most caregivers incurred medical expenses (34.2%). Additionally, 76.9% of caregivers expressed interest in the hypothetical RSV vaccination. **Conclusions:** In Japan, caregivers of infants with RSV experience had significant psychological burden regardless of whether the treatment is outpatient or inpatient. In addition, a non-negligible proportion of caregivers suffer from societal and economic burdens. This study lays the groundwork for all stakeholders to fully comprehend the comprehensive disease burden of child RSV infections.

## 1. Introduction

Respiratory syncytial virus (RSV) causes seasonal respiratory infections and is a leading cause of acute lower respiratory tract infections (LRTI) in infants and young children [1]. It is estimated to account for approximately 50% of pneumonia and 50–90% of bronchiolitis cases in infants [2]. Infants with bronchioles with an inner diameter of 120 μm, approximately half that of adults, are more susceptible to bronchiolitis and respiratory complications from RSV infection [3]. The disease is particularly severe if the infection occurs before the age of 6 months [4]. In Japan, approximately 120,000–140,000 children under the age of two are diagnosed with RSV infection annually, with one-quarter of these cases (approximately 30,000) requiring hospitalization [5].

In recent years, the landscape of RSV prevention has rapidly evolved with the introduction of newly approved vaccines and monoclonal antibodies aimed at preventing RSV infections in infants and older adults [6,7]. In order to make informed decisions, stakeholders must understand the disease burden of RSV, since awareness among patients, policymakers, and healthcare providers remains low at present [6]. A comprehensive examination of the burden of RSV infection is incomplete without evaluating its impact on caregivers, family members caring for children, and the direct and indirect costs to society. While several studies have reported significant economic and psychological burdens of RSV infection in children on caregivers [8,9], many have focused on high-risk populations for severe RSV LRTI, including infants with underlying diseases, preterm birth, or hospitalized patients [10], preventing us from gaining a holistic view. Furthermore, the burden of RSV infection on caregivers may vary across societies, owing to historical, societal, and cultural differences that affect how children and families experience the disease. Each society can also have unique public health policy priorities based on its circumstances, such as finite resources and demographics. Despite its public health importance, the burden on caregivers of patients with RSV infections in Japan remains uncertain.

To address this knowledge gap, we investigated the psychological, social, and economic burdens on caregivers when infants aged <1 year are infected with RSV in Japan. 

## 2. Materials and Methods

### 2.1. Study Design

We conducted a non-interventional, cross-sectional study from 16 to 31 January 2023. Participants were recruited using the Automatic Internet Research System, an online platform provided by Macromill Carenet, Inc. (Tokyo, Japan). The company has more than 1.3 million registered members from all prefectures in Japan, with diverse sociodemographic characteristics (e.g., both men and women, a wide range of age groups, and occupational statuses). Participants had the option to discontinue or terminate the survey at any time without providing a reason. The survey system did not permit participants to proceed to the next page if there were unanswered items. The study was designed without treatment restrictions and did not involve the recording or evaluation of adverse events. 

### 2.2. Research Participants

The study participants were caregivers of children infected with RSV who met the following criteria: (1) caregivers of infants infected with RSV at 11 months of age or younger in 2023 (registered caregivers whose infants were under 1 year of age at the time of RSV infection diagnosis); (2) caregivers who cared for RSV-infected children; (3) caregivers who lived with RSV-infected children at the time of infection; (4) caregivers who could access the dedicated web system and answer questions from an Internet-connected device (their own computer, smartphone, or other); (5) caregivers who provided appropriate consent to participate in this study through the dedicated web system. Regarding the RSV infection diagnosis, during the screening process, potential participants were explicitly asked whether their children were diagnosed with laboratory-confirmed RSV infection in 2023, and only those who answered “yes” proceeded to the actual survey.

Caregivers who met the following criteria were excluded from the study: (1) under 20 years of age at the time of obtaining consent; (2) did not provide consent; (3) registered outside of the registration period.

The target number of caregivers surveyed in this study was 500. With the aim of examining differences based on infants’ age at the time of RSV infection, we enrolled at least 250 caregivers of infants under 6 months of age and 250 caregivers of infants between 6 and 12 months of age, ensuring a similar number of infants in each group. The number of cases was determined based on feasibility, rather than hypothesis testing.

### 2.3. Measures

Sociodemographic and clinical data were collected from both infants and caregivers. Caregivers completed multiple study measures. The following background factors were summarized: for infants with RSV, age in months at the time of consent and RSV infection; for caregivers, sex, age, annual household income, marital status, working hours, and parental involvement in infant care at the time of RSV infection. Additionally, we collected data on the clinical characteristics of children with RSV infection, including the use of palivizumab prophylaxis, complications, symptoms, hospitalization, length of hospitalization, and treatment during hospitalization.

The primary endpoint was the psychological burden on caregivers due to infant RSV infection. Secondary endpoints were as follows: (1) social burden on caregivers due to RSV infection in infants (impact on daily life and social activities); (2) impact of infant RSV infection on caregivers’ economic and working environment (effect on working hours and working conditions); (3) caregivers’ preferences regarding RSV prophylaxis (route of administration). To assess the preferences for RSV prophylaxis, information on RSV and hypothetical RSV prophylaxis was provided.

In the case of caregivers responsible for several infants infected with RSV, the youngest infant was referenced in their responses. In addition, if the infant had several RSV episodes, caregivers responded in relation to the first RSV episode.

### 2.4. Data Handling and Statistical Analyses

Data collected from the study participants through a dedicated web system were anonymized by Macromill Carenet Inc. before being provided to the research office. All statistical analyses were conducted using SAS 9.4 (SAS Institute, Cary, NC, USA) or R4.1.2. (R Foundation for Statistical Computing, Vienna, Austria).

Participants who were enrolled in this study and answered at least one item were considered part of the full analysis set (FAS). Summary statistics for each item were calculated. Frequencies and proportions were calculated for categorical variables, whereas the number of cases, mean value, standard deviation, median value, interquartile range (Q1 and Q3), maximum value, and minimum value were calculated for continuous variables. Additionally, a logistic regression analysis was conducted to determine whether there were differences in the psychological burden on caregivers depending on the infant’s age at the time of RSV infection. In this analysis, the question about whether parents experienced anxiety regarding their infants infected with RSV in the parental psychological burden questions served as the outcome, and the five-point Likert scales were divided into two categories: agree and disagree. Infant age at the time of infection (<6 months) was used as the explanatory variable (the main effect). In addition, the following variables were used as covariates: parent’s sex, age, presence of family members living together, parent’s involvement in infant care, infant’s birth weight, gestational week, infant’s sex, presence or absence of risk factors for severe disease (congenital heart disease (1), Down’s syndrome (2), immunodeficiency (3), bronchial asthma (4), underlying lung disease (5), passive smoke (6), exposure to Human Immunodeficiency Virus (7), artificial milk (8), or none of the above (9), use of childcare facilities for the infant, employment status of parents, and level of knowledge regarding RSV infection.

### 2.5. Ethical Considerations

The study protocol was approved by the Ethics Review Committee (Shiba Palace Clinic Ethics Review Committee) on 14 December 2023. This study adhered to the principles outlined in the World Medical Association Declaration of Helsinki (revised in 2013) and the Ethical Guidelines for Life Science and Medical Research Involving Human Subjects (revised on 10 March 2020, by the Ministry of Education, Culture, Sports, Science and Technology/Ministry of Health, Labour and Welfare, Ministry of Economy, Trade and Industry), along with other pertinent laws and regulations.

During the process of obtaining consent from the study participants, all necessary information for individuals to make informed decisions regarding their participation in the research was presented on the web system. Consent was considered appropriate when the participant pressed the consent button, indicating agreement to participate. Participation consent data were securely stored by Macromill Carenet Inc., along with the questionnaire survey data, ensuring confidentiality and data security.

## 3. Results

### 3.1. Selection of Participating Caregivers

**Figure 1:**
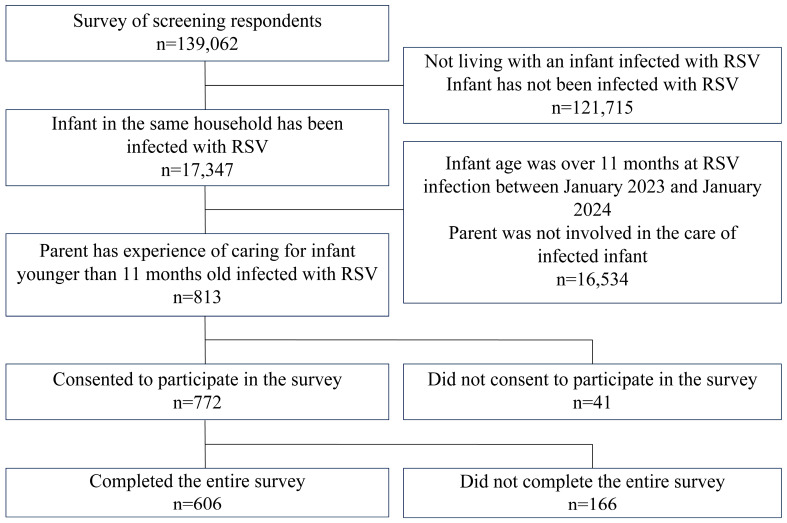
Flow of participants included in the study.

This study used questionnaire responses collected from 16 January 2023 to 31 January 2023. A total of 139,062 participants were screened during the study period. Among them, 121,715 cases were excluded because the infants had no history of RSV infection, and 16,534 cases were excluded because either the infant was not younger than 11 months of age at the time of RSV infection between January 2023 and January 2024 or the caregivers were not involved in caring for the infant (Figure 1). From the remaining pool of 813 caregivers who were involved in caring for infants aged 11 months or younger at the time of RSV infection, a questionnaire survey was administered to 772 caregivers who consented to participate in the study. The final analysis included responses from 606 participants constituting the FAS, which were subsequently analyzed.

### 3.2. Characteristics of Surveyed Infants with RSV and Their Caregivers

Table 1 presents the data of 606 infants with RSV, along with their caregivers and circumstances during the RSV infection period. Equal numbers of infants aged under 6 months and over 6 months were enrolled, and infant age at infection was distributed as follows: ≥0 to <3 months (33.0%), ≥3 to <6 months (17.0%), and ≥6 to <12 months (50.0%). Approximately 10.6% of the infants had a low birth weight (<2500 g), and 17.7% were born preterm (<37 weeks of gestation).

Most of the questionnaire respondents were female (78.9%), with an average age (SD) of 34.2 (5.6) years, and most were married (95.4%). Most of the respondents primarily cared for RSV-infected children (89.9%). Regarding employment status, half of the caregivers were employed full time (41.4%) or short time/part time (11.2%), 28.7% were on maternity/childcare leave, and 18.3% were unemployed. Approximately 70.5% of the infants did not receive palivizumab prophylaxis at the time of RSV infection. Some patients had underlying conditions, such as infant congenital heart disease (2.0%), bronchial asthma (6.3%), and exposure to secondhand smoke (9.4%). The main symptoms of RSV infection included cold-like symptoms, such as runny nose (81.7%), fever (80.4%), and cough (78.1%). However, severe respiratory symptoms, such as dyspnea (13.7%) and apnea (3.1%), were also observed. Approximately 36.1% of infants were hospitalized, with nearly 70% of hospitalizations lasting <10 days (≤6 days: 36.5%, 7–9 days: 32.9%). However, 0.9% of infants were hospitalized for more than one month, and 7.3% of infants were still hospitalized. During hospitalization, the main treatments included drip management (74.0%), inspiration (69.9%), internal medication (68.0%), and oxygen therapy (60.3%). ICU (5.0%) and ventilatory management (4.6%) were also used. Approximately half of the infants (46.5%) were diagnosed with RSV infection alone, and diagnosed symptoms and complications of RSV infection included bronchitis/bronchiolitis (36.1%) and upper respiratory tract infection (17.2%).

### 3.3. Psychological Burden of RSV Infection in Infants on the Caregivers

For the primary endpoint of psychological burden, the mental status of the caregivers was investigated. Among all caregivers, 17.0% of caregivers were extremely worried about their infant’s RSV infection interfering with their daily life, among whom 38.8% said their worry interfered with their daily life, and 35.6% said they were worried, but their worry did not interfere with their daily life (Table 2). In other words, 91.4% of caregivers felt anxious about their infant’s RSV infection, and more than half (55.8%) answered that their anxiety interfered with their daily lives. A higher percentage of female caregivers (60.9%) than male caregivers (36.7%) indicated that anxiety about their infant’s RSV infection symptoms interfered with their daily lives. Similarly, a higher tendency was observed among caregivers whose infants were hospitalized due to RSV infection (67.1%) than among caregivers whose infants were not hospitalized (49.4%) about their infant’s RSV infection symptoms had interfered. Looking at the length of hospitalization, 79.7% of caregivers who were hospitalized for a long period of time (≥7 days) answered that their anxiety interfered with their daily lives. This rate tended to be higher than that of the 57.5% of caregivers whose hospitalizations were shorter (57.5%). Regarding the age of infants with RSV, the number of caregivers who answered that their anxiety interfered with their daily lives was the same for infants under 6 months (54.1%) and those aged 6–12 months (57.4%).

**Figure 2:**
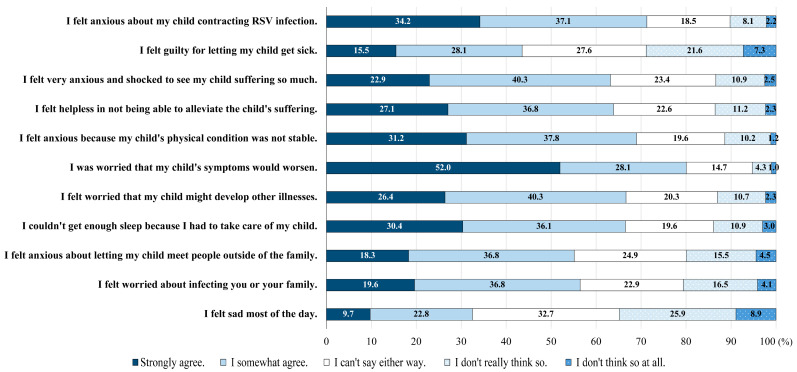
Emotional burden of caregivers.

Upon closer examination of caregivers’ concerns regarding infant RSV infection, 80.1% of caregivers strongly agreed or somewhat agreed with worry about their infant’s worsening symptoms, while 71.3% strongly agreed or somewhat agreed with worry about you and your family being infected with RSV. (Figure 2). Among other anxiety factors, the most prevalent included worries about the infant’s physical condition not stabilizing (69.0%) and concerns about the possibility of other diseases occurring simultaneously (66.7%). A significant proportion of caregivers (66.5%) reported insufficient sleep due to late-night nursing care and many (63.9%) expressed feelings of powerlessness in alleviating their infants’ suffering. Additionally, 43.6% of caregivers experienced feelings of guilt about potentially infecting their infants. Conversely, 28.9% of the caregivers indicated that they did not experience such emotions or thoughts.

Multivariate analysis (Table 3) was conducted to investigate the potential differences in the psychological burden experienced by caregivers based on the infant’s age at the time of RSV infection. The odds ratio for caregivers reporting anxiety when their infant was <6 months old compared with when their infant was ≤6–<12 months old was 1.43 (95% confidence interval [CI]: 0.95–2.16, *p*-value = 0.083), indicating no statistically significant difference.

Other variables deemed confounders included the sex and age of the caregiver, family members living with the caregiver, involvement of the caregiver in the care of infants, birth weight of infants, gestational age at infant birth, and infant sex. Risk factors for severe disease were as follows: congenital heart disease (1), Down’s syndrome (2), immunodeficiency (3), bronchial asthma (4), underlying lung disease (5), exposure to secondhand smoke (6), exposure to human immunodeficiency virus (7), artificial milk (8), or none of the above (9), as well as use of childcare facilities for RSV-infected infants, employment status of caregivers, and knowledge about RSV.

### 3.4. Impact of Infant with RSV Infection on Caregivers’ Daily Lives

**Figure 3:**
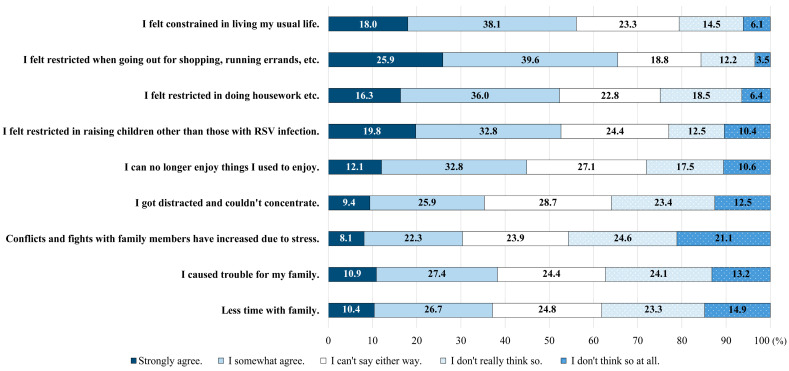
Societal burden on caregivers.

Approximately 56.1% of caregivers (strongly agree: 18.0%; somewhat agree: 38.1%) reported that their infants’ RSV infections disrupted their usual routines. Additionally, 65.5% of caregivers felt limited in their ability to go out for shopping or errands (strongly agree: 25.9%; somewhat agree: 39.6%). Most caregivers (52.6%; strongly agree: 19.8%; somewhat agree: 32.8%) perceived restrictions on childcare for non-infected children. Moreover, 35.3% of caregivers (strongly agree: 9.4%, somewhat agree: 25.9%) expressed difficulty concentrating because of distractions, while 44.9% (strongly agree: 12.1%, somewhat agree: 32.8%) reported a loss of enjoyment in their usual activities. Furthermore, 37.1% of caregivers (strongly agree: 10.4%, somewhat agree: 26.7%) cited spending less time with their family, and 30.4% (strongly agree: 8.1%, somewhat agree: 22.3%) noted increased stress, leading to conflicts and disputes among family members (Figure 3).

### 3.5. Impact of Infant with RSV Infection on Caregivers’ Economic and Working Environment

Regarding the economic burden of RSV infection in infants, the most common expenditure reported by caregivers was “medical expenses” (34.2%). This was followed by “Items that cost more than the usual food expenses for the entire household” (28.2%), “transportation expenses for treatment” (19.3%), and “Hospitalization costs (different bed charges, etc.)” (17.8%) (Table 4). Among the 391 caregivers who indicated experiencing financial strain, excluding subsidies for medical expenses, 56.0% reported spending JPY < 30,000 (JPY < 10,000, 36.8%, JPY 10,000–30,000: 19.2%). However, 14.6% of the caregivers faced a financial burden ranging from JPY 100,000 to 200,000 (Table 4). Regarding the impact on work, 85.4% of caregivers and 46.7% of their partners reported job-related disruptions owing to their child’s RSV infection. Among the 321 caregivers who were employed at the time of the child’s RSV infection, 37.7% took paid leave, averaging (SD) 4.3 (3.6) days off, and 44.2% reduced their working hours by leaving work early. Similarly, among the respondents’ partners (469 cases), 17.3% took paid leave, whereas 26.9% reduced their working hours by leaving early (Table 4).

### 3.6. Caregivers’ Preferences for RSV Prophylaxis (Route of Administration)

If the RSV vaccine was available, 76.9% of the caregivers expressed their willingness to use it for their children or pregnant women (Table 5). Regarding the target population for vaccination, among all respondents, the preference for vaccinating infants (38.0%) was comparable to that for pregnant women (38.9%). However, a higher proportion of caregivers of infants under 6 months of age who had been infected with RSV (43.6%) expressed a preference for vaccinating pregnant women than caregivers of infants aged 6–12 months (34.3%). Additionally, caregivers of infants who experienced severe respiratory symptoms such as apnea or dyspnea due to RSV infection were more inclined to wish to vaccinate pregnant women (47.8%) than their infants (31.9%). Although the number of infants placed on ventilators or admitted to the ICU during hospitalization was small (15 cases), a larger percentage of caregivers expressed a preference for vaccinating pregnant women (66.7%) over vaccinating infants (26.7%). There was a discernible trend indicating that the higher the knowledge level regarding the disease, the greater the proportion of caregivers who preferred to vaccinate pregnant women rather than infants. Among caregivers with the highest level of knowledge, the desire to vaccinate pregnant women (49.8%) exceeded that for infants (25.5%).

## 4. Discussion

In Japan, research on infants with RSV infection has primarily focused on disease morbidity [5] and trends in epidemiology and treatment patterns [11]. However, there is a notable lack of research addressing the psychological burden experienced by these infats’ caregivers and its consequences. This study aimed to fill this research gap by focusing on the psychological, social, and economic burdens faced by caregivers of children infected with RSV. Consequently, this study represents the first attempt to directly survey the caregivers of infants who have experienced RSV infection, with over 600 participants across Japan.

The results of this study revealed that, in Japan, >90% of caregivers experience anxiety when their infants contract RSV, with over half indicating that this anxiety disrupts their daily lives. Reports from other countries have highlighted that the hospitalization of infants and young children due to RSV infection leads to decreased quality of life (QOL) [12] and induces severe stress in caregivers [8,13]. Moreover, a European cohort study [14] focusing on infants infected with RSV, even if they did not require medical attention, found that approximately 60% of caregivers reported an impact on their daily lives. Thus, regardless of the severity of the infection, caregivers of Infants with RSV are likely to experience anxiety and significant disruptions in their daily routines. The present study included infants treated both in outpatient settings and inpatient settings for RSV infection, revealing that the majority of caregivers experienced significant anxiety, leading to substantial disruptions in their daily lives. Consistent with the findings from other countries’ reports, caregivers of infants infected with RSV face a considerable burden in Japan. Additionally, 42.4% of the infants hospitalized with RSV in Japan were younger than 6 months of age. Moreover, previous research has indicated that 50% of the infants requiring mechanical ventilation during hospitalization for RSV infection are under 6 months of age [5], suggesting a higher likelihood of severe illness among infants in this age group when infected with RSV. Hence, it was hypothesized that caregivers of infants under 6 months of age, who are at a higher risk of severe illness, would experience greater anxiety. Accordingly, multivariate analysis was conducted to assess the impact of infant age at the time of RSV infection on parental anxiety. Although parental anxiety tended to be higher when infants were <6 months old than when those were 6–12 months, no statistically significant differences were observed. However, some overseas reports [12] have suggested that infant age at the time of RSV infection does not significantly affect parental QOL. Hence, further investigation is warranted to explore the influence of infant age on parental anxiety.

The anxiety and psychological burden experienced by caregivers include concerns about the deterioration of their infant’s symptoms, anxiety about contracting an RSV infection, and uncertainty regarding the duration of recovery. Moreover, along with worries about the infant’s health, such as the possibility of complications, caregivers experienced personal burdens, including disruptions in their sleep patterns and a sense of helplessness in alleviating their infant’s distress. Examining the challenges to daily life, the most prevalent issue was the limitation of household errands or outings. However, a notable proportion of respondents also reported heightened conflict and tension among family members due to stress and childcare constraints for non-infected children. These findings align with those of prior research [8,9], indicating that the stress experienced by patients and their caregivers persists not only during infection episodes but also after discharge. A US study focusing on hospitalized patients with RSV infection suggested that the disruption caused by unexpected RSV infection and hospitalization of a child could significantly affect other family members and their daily routines. Based on insights from previous studies targeting preterm infants at high risk for severe RSV infection [8,9,13], this research extends the evidence base by highlighting that even common RSV infections, including those managed in outpatient settings, impose diverse psychosocial burdens on caregivers.

We also examined the economic burden on the caregivers who take care of infants with RSV infection. In many local governments in Japan, children’s medical expenses are either fully covered or kept at minimal cost through a universal health insurance system and subsidies for children’s medical expenses [15,16]. The specifics of the subsidy system vary across regions. For instance, in Tokyo, medical and meal expenses during hospitalization are subsidized in some wards. Consequently, the out-of-pocket expenditure for medical care in Japan is lower than that in other countries, such as the average out-of-pocket expense for infants hospitalized with RSV infection in Canada (CAD 736.69) [13]. However, half of the caregivers in our study still incurred out-of-pocket expenses of JPY < 30,000 [~USD 214 (JPY/USD 140)]. In addition, a recent study utilizing a large health insurance claims database in Japan [11] reported that the average medical cost for hospitalizing infants under the age of five with RSV infection was JPY 510,000 per inpatient [~USD 3643]. These costs are still lower than those reported in other industrialized countries, such as USD 14,823 per hospitalization in the US and SEK 131,997 per hospitalization in Sweden [17,18]. Additionally, in our survey, approximately 40% of caregivers and partners took paid leave or reduced their working hours to care for infants with RSV infection. Similarly, a report from other countries noted that caregivers of infants hospitalized with RSV infection had an average absenteeism rate of 49.0% and an overall work disability rate of 77.8% [13]. Even among caregivers of infants who did not seek medical attention, the absentee rate was 10.8% [14]. These findings underscore the socioeconomic implications of RSV infection in infants, including reduced productivity and economic losses, that cannot be overlooked.

In this study, from the experiences of RSV infection in infants aged <1 year, we conducted a survey of caregivers’ preferences regarding a hypothetical RSV vaccine, based on their experiences with RSV infection in infants aged <1 year. Approximately three-quarters of the caregivers expressed a preference for vaccination. Particularly noteworthy were the preferences of caregivers of infected infants under 6 months of age, those whose infants experienced severe respiratory symptoms, such as apnea or dyspnea, and those whose infants were placed on a ventilator or admitted to the ICU. In these groups, there was a tendency to favor the vaccination of pregnant women over infants. Additionally, caregivers with extensive prior knowledge of RSV infection were more likely to be in favor of vaccinating pregnant women compared to infants. The reason for this tendency remains unclear. However, it appears that, based on their first-hand experiences and understanding of the potential severity of RSV infection in infants, caregivers’ preference to prioritize the vaccination of pregnant women has increased.

After concluding the data collection for the study, two new preventive measures for a respiratory syncytial virus (RSV)-associated lower respiratory tract infection (LRTI) in infants have been approved in Japan: a single dose of Pfizer’s RSV vaccine (Abrysvo) and nirsevimab (Beyfortus, Sanofi and AstraZeneca), a long-acting monoclonal antibody. The Japan Pediatric Society and Japan Society of Obstetrics and Gynecology have issued statements for their fellows [19,20,21]. These approvals are in line with multiple countries and have already been incorporated into the National Immunization Program in some countries [22,23,24], with reports beginning to emerge on the impact of reducing disease burden in the 2023–2024 season [25,26]. Due to the burden of RSV infection not only on children but also on families and society as a whole, clinicians are recommended to counsel pregnant individuals about the maternal RSV vaccine and the monoclonal antibody, nirsevimab, as safe and effective ways to prevent severe LRTI caused by RSV in infants, as recommended by The American College of Obstetricians and Gynecologists (ACOG) [27]. 

This study has several inherent limitations. First, this study relied on reports by caregivers lacking direct verification of the RSV diagnosis, which introduces the possibility of information bias, potentially influencing the results. In addition, there is a risk of recall bias. Second, selection bias could be a concern if survey participation is correlated with the burden of RSV infection experienced by caregivers. Third, although this study included caregivers registered in the MCN panel, the findings may not be generalizable to a broader population. Nonetheless, most previous reports on infant RSV infection were confined to studies involving high-risk groups, such as preterm infants and those with underlying diseases. In contrast, this survey’s examination of the psychological, social, and economic burdens encompassed the general patient population, including outpatients and those with milder cases, underscoring its significance.

## 5. Conclusions

A questionnaire survey was conducted across Japan among 606 caregivers who cared for infants with RSV infections. Based on the results, it is evident that caregivers not only face a psychological burden when their infants are hospitalized or in outpatient care, but that infection also has a significant social and economic impact. Three-quarters of the caregivers of infants with RSV indicated that they would prefer to vaccinate their children or pregnant women in the future. Pregnant women were more likely to request vaccination for themselves than for their infants if the infected infant was <6 months old or had severe disease (i.e., severe respiratory symptoms, ventilatory management, or ICU admission).

## Figures and Tables

**Table 1:** Characteristics of respiratory syncytial virus (RSV)-infected infants and caregivers.

No. of Responders (%)			All
			n = 606
Infants with RSV	Sex	Male	380 (62.7%)
		Female	226 (37.3%)
	Infant age at infection (months)	≥0 to <6 months	303 (50.0%)
		≥0 to <3 months	200 (33.0%)
		≥3 to <6 months	103 (17.0%)
		≥6 to <12 months	303 (50.0%)
	Birth weight	<2500 g	64 (10.6%)
		≥2500 g	542 (89.4%)
	Gestational age at infant’s birth	<37 weeks	107 (17.7%)
		≥37 weeks	499 (82.3%)
	Use of childcare facilities for RSV-infected infant	Yes	257 (42.4%)
		No	349 (57.6%)
Caregiver	Age	Mean (SD)	34.2 (5.6)
		Median (Q1, Q3)	34.0 (30.0, 37.0)
		Min, max	20,57
		20–24 years	12 (2.0%)
		25–29 years	111 (18.3%)
		30–34 years	208 (34.3%)
		35–39 years	174 (28.7%)
		40–44 years	69 (11.4%)
		45–49 years	24 (4.0%)
		50–54 years	6 (1.0%)
		55–59 years	2 (0.3%)
		≥60 years	0 (0%)
	Sex	Male	128 (21.1%)
		Female	478 (78.9%)
	Annual household income (JPY)	<2,000,000	9 (1.5%)
		2,000,000–4,000,000	77 (12.7%)
		4,000,000–6,000,000	105 (17.3%)
		6,000,000–8,000,000	92 (15.2%)
		8,000,000–10,000,000	47 (7.8%)
		10,000,000–12,000,000	30 (5.0%)
		12,000,000–15,000,000	75 (12.4%)
		15,000,000–20,000,000	7 (1.2%)
		≥20,000,000	8 (1.3%)
		Unknown	48 (7.9%)
		Not stated	108 (17.8%)
	Marital status at the time of RSV infection	Married	578 (95.4%)
		Separated or bereaved	19 (3.1%)
		Unmarried	9 (1.5%)
	Involvement of caregiver in the care of infant	Primary caregiver (mainly cared for the infant)	545 (89.9%)
		Someone else did the primary nursing care.	61 (10.1%)
		Not involved in nursing at all.	0 (0%)
	Employment status of caregiver	Full-time work (work in the office at least 3 days a week)	230 (38.0%)
		Full-time work (work from home at least 3 days a week)	21 (3.5%)
		Short time/part time	68 (11.2%)
		During maternity or childcare leave	174 (28.7%)
		Other	2 (0.3%)
		Unemployed	111 (18.3%)
Characteristics of RSV-infected infant	Use of palivizumab prophylaxis against RSV infection	Never administered	427 (70.5%)
		Palivizumab previously administered, discon-tinued prior to the RSV infection onset	56 (9.2%)
		Receiving palivizumab prophylaxis at the on-set of RSV infection	34 (5.6%)
		Don’t know/don’t remember	89 (14.7%)
	Risk factor for severe disease ***	Diagnosed with infant congenital heart disease	12 (2.0%)
		Diagnosed with Down syndrome	2 (0.3%)
		Diagnosed with immunodeficiency (severe combined immunodeficiency, leukemia, hematopoietic cell/lung transplantation, etc.)	7 (1.2%)
		Diagnosed with bronchial asthma	38 (6.3%)
		Diagnosed with a lung disease such as chronic lung disease (bronchopulmonary dysplasia) or cystic fibrosis	7 (1.2%)
		Infant may be exposed to second-hand smoke (Infant may be in an area where someone is smoking in your home or car)	57 (9.4%)
		Exposed to human immunodeficiency virus (someone close to you, such as your mother, has been infected)	11 (1.8%)
		Childcare consisted mainly of artificial milk (powdered milk, etc.)	147 (24.3%)
		None of the above apply	394 (65.0%)
	Symptom ***	Sneezing	209 (34.5%)
		Runny nose	495 (81.7%)
		Fever	487 (80.4%)
		Wheezing (breathing sounds such as whistling and wheezing)	265 (43.7%)
		Cough	473 (78.1%)
		Apnea (including feeling like you are about to stop breathing)	19 (3.1%)
		Dyspnea (breathing seems difficult, shortness of breath, etc.)	83 (13.7%)
		Sleeplessness	132 (21.8%)
		Decrease in suckling amount, decrease in food intake	165 (27.2%)
		Decreased urination volume	42 (6.9%)
		Irritability	141 (23.3%)
		Cramps	11 (1.8%)
		Paleness	37 (6.1%)
		Others	17 (2.8%)
		Don’t know/don’t remember	5 (0.8%)
	Hospitalization	Hospitalized	219 (36.1%)
		Not hospitalized	387 (63.9%)
	Duration of hospitalization ***	Within 3 days	20 (9.1%)
		4–6 days	60 (27.4%)
		7–9 days	72 (32.9%)
		10–14 days	45 (20.5%)
		15–30 days	4 (1.8%)
		>31 days	2 (0.9%)
		Still under treatment and not fully recovered	16 (7.3%)
	Type of treatment during hospitalization *,***	Inspiration	153 (69.9%)
		Internal medication (e.g., expectorants)	149 (68.0%)
		Oxygen therapy (oxygen mask, oxygen hood, etc., or oxygen administration for less severe cases than ventilation)	132 (60.3%)
		Drip management (intravenous infusion, etc.)	162 (74.0%)
		Antibacterial (antibiotics)	82 (37.4%)
		Steroid	27 (12.3%)
		Ventilatory management (use of ventilators in hospitalization, etc.)	10 (4.6%)
		Intensive care unit management (ICU)	11 (5.0%)
		Other (FA)	3 (1.4%)
		Don’t know/don’t remember	2 (0.9%)
		Ventilatory management or ICU **	15 (6.9%)
	Complication ***	Upper respiratory tract infection (cold symptoms)	104 (17.2%)
		Bronchitis (bronchiolitis)	219 (36.1%)
		Pneumonia	42 (6.9%)
		Otitis media	46 (7.6%)
		Febrile seizure	22 (3.6%)
		Encephalopathy/encephalitis	4 (0.7%)
		Other	5 (0.8%)
		No other diagnosis besides RSV infection	282 (46.5%)

*: The denominator is hospitalized cases (n = 219) **: Includes six patients who were admitted to the ICU and underwent ventilatory management. ***: This question had multiple answers, except none of the above/Don’t know/don’t remember / No other diagnosis besides RSV infection.

**Table 2:** Psychological and social burden on caregivers. Answers to the question: “How did you feel when your child contracted RSV infection?”

Answer		All	Sex of Caregiver		Hospitalization		Duration of Hospitalization or Disease		Infant Age at Infection	
			Male	Female	Hospitalized	Not Hospitalized	≤6 days	≥7 days	≥0 to <6 months	≥6 to <12 months
		n = 606	n = 128	n = 478	n = 219	n = 387	n = 80	n = 123	n = 303	n = 303
Didn’t interfere with your life		255 (42.1%)	73 (57.0%)	182 (38.1%)	69 (31.5%)	186 (48.1%)	32 (40.0%)	24 (19.5%)	133 (43.9%)	122 (40.3%)
	You didn’t worry too much about your child and it didn’t interfere with your life.	39 (6.4%)	22 (17.2%)	17 (3.6%)	15 (6.8%)	24 (6.2%)	5 (6.3%)	4 (3.3%)	22 (7.3%)	17 (5.6%)
	You were worried about your child, but it didn’t interfere with your life.	216 (35.6%)	51 (39.8%)	165 (34.5%)	54 (24.7%)	162 (41.9%)	27 (33.8%)	20 (16.3%)	111 (36.6%)	105 (34.7%)
Interfered with your life		338 (55.8%)	47 (36.7%)	291 (60.9%)	147 (67.1%)	191 (49.4%)	46 (57.5%)	98 (79.7%)	164 (54.1%)	174 (57.4%)
	Worrying about your child has interfered with your life.	235 (38.8%)	28 (21.9%)	207 (43.3%)	96 (43.8%)	139 (35.9%)	21 (26.3%)	74 (60.2%)	106 (35.0%)	129 (42.6%)
	You were extremely worried about your child and it interfered with your daily life.	103 (17.0%)	19 (14.8%)	84 (17.6%)	51 (23.3%)	52 (13.4%)	25 (31.3%)	24 (19.5%)	58 (19.1%)	45 (14.9%)
I don’t know/I don’t remember.		13 (2.1%)	8 (6.3%)	5 (1.0%)	3 (1.4%)	10 (2.6%)	2 (2.5%)	1 (0.8%)	6 (2.0%)	7 (2.3%)

**Table 3:** Multivariate analysis: Effect of infant’s age on caregiver’s psychological burden.

Infant Age at RSV Infected	Odds Ratio	95% CI	*p*-Value
≥6 to <12 months	(reference)		
<6 months	1.43	0.95–2.16	0.083

**Table 4:** Impact of infant RSV infection on caregivers’ economic and working environment.

Economic burden of infant RSV infection *		
		All
		n = 606
They had a financial burden.		391 (64.5%)
Medical expenses		207 (34.2%)
Transportation expenses for treatment		117 (19.3%)
Sick childcare (service to care for and care for sick children)		42 (6.9%)
Sitter fee (excluding children with RSV infection)		26 (4.3%)
Items that cost more than the usual food expenses for the entire household		171 (28.2%)
Hotel expenses for the caregiver or person being cared for		21 (3.5%)
Hospitalization costs (different bed charges, etc.)		108 (17.8%)
Others		17 (2.8%)
There was no financial burden.		215 (35.5%)
	
Medical expense burden		
		There was financial burden
		n = 391
JPY < 10,000		144 (36.8%)
JPY 10,000–30,000		75 (19.2%)
JPY 30,000–50,000		33 (8.4%)
JPY 50,000–100,000		23 (5.9%)
JPY 100,000–200,000		57 (14.6%)
JPY 200,000–300,000		30 (7.7%)
JPY 300,000–400,000		4 (1.0%)
JPY 400,000–500,000		3 (0.8%)
JPY 500,000–1 million		3 (0.8%)
JPY 1 million–2 million		2 (0.5%)
JPY ≥ 2 million		0 (0%)
I don’t know/I don’t remember		17 (4.4%)
	
Impact of RSV infection on work in infants †		
	Caregivers	Spouse/partner
	n = 321	n = 469
I took paid leave.	121 (37.7%)	81 (17.3%)
I took unpaid leave.	24 (7.5%)	6 (1.3%)
Reduced working hours by leaving work early etc.	142 (44.2%)	126 (26.9%)
I had someone else follow up on my work.	64 (19.5%)	50 (10.7%)
I quit my job	2 (0.6%)	3 (0.6%)
Others	3 (0.9%)	5 (1.1%)
There was no particular impact.	47 (14.6%)	250 (53.3%)

* This question had multiple answers except “There was no financial burden”. † This question had multiple answers except “There was no particular impact”.

**Table 5:** Parental preferences for RSV prophylaxis (route of administration).

Answer	All	Infant Age at Infection		Symptoms of Infant *			Type of Treatment during Hospitalization		Knowledge about RSV Infection		
		0–6 Months	6–12 Months	(A)	I Don’t Know/I Don’t Remember (B)	Other than(A)(B)	Ventilatory Management or ICU	No Ventilatory Management or ICU	I Had Never Heard of It **	I Had Heard of It, but I Didn’t Know What Kind of Disease It Was.	I Had Heard of it and Had Some Idea of What Kind of Disease It Was.
	n = 606	n = 303	n = 303	n = 276	n = 5	n = 325	n = 15	n = 591	n = 116	n = 247	n = 243
I would like to vaccinate	466 (76.9%)	226 (74.6%)	240 (79.2%)	220 (79.7%)	3 (60.0%)	243 (74.8%)	14 (93.3%)	452 (76.5%)	82 (70.7%)	201 (81.4%)	183 (75.3%)
I would like to vaccinate my child after giving birth.	230 (38.0%)	94 (31.0%)	136 (44.9%)	88 (31.9%)	1 (20.0%)	141 (43.4%)	4 (26.7%)	226 (38.2%)	47 (40.5%)	121 (49.0%)	62 (25.5%)
I would like to have the mother vaccinated during pregnancy ***	236 (38.9%)	132 (43.6%)	104 (34.3%)	132 (47.8%)	2 (40.0%)	102 (31.4%)	10 (66.7%)	226 (38.2%)	35 (30.2%)	80 (32.4%)	121 (49.8%)
I don’t want either	61 (10.1%)	38 (12.5%)	23 (7.6%)	25 (9.1%)	1 (20.0%)	35 (10.8%)	0 (0%)	61 (10.3%)	16 (13.8%)	17 (6.9%)	28 (11.5%)
I don’t know	79 (13.0%)	39 (12.9%)	40 (13.2%)	31 (11.2%)	1 (20.0%)	47 (14.5%)	1 (6.7%)	78 (13.2%)	18 (15.5%)	29 (11.7%)	32 (13.2%)

* Symptoms of infant (A): apnea (including feeling like you are about to stop breathing), dyspnea (breathing seems difficult, shortness of breath, etc.), inability to sleep, decrease in suckling amount, decrease in food intake, decreased urination volume, cramps, and looks pale. ** I had never heard of it (I only learned about it after my child was diagnosed). *** I would like to have the mother vaccinated during pregnancy (by giving the mother the vaccine, the mother’s antibodies will be transferred to the child and protect them from infection).

## Data Availability

The datasets presented in this article are not readily available because, owing to the nature of this research, the participants in this study did not agree that their data would be publicly shared. However, the data may be available from the authors upon reasonable request.
